# Maternal Diabetes and Overweight and Congenital Heart Defects in Offspring

**DOI:** 10.1001/jamanetworkopen.2023.50579

**Published:** 2024-01-05

**Authors:** Riitta Turunen, Anna Pulakka, Johanna Metsälä, Tero Vahlberg, Tiina Ojala, Mika Gissler, Eero Kajantie, Emmi Helle

**Affiliations:** 1 Pediatric Research Center, New Children’s Hospital, Helsinki University Hospital and University of Helsinki, Helsinki, Finland; 2 Population Health Unit, Finnish Institute for Health and Welfare, Helsinki, Finland; 3 Research Unit of Population Health, Faculty of Medicine, University of Oulu, Oulu, Finland; 4 Department of Biostatistics, Faculty of Medicine, University of Turku, Turku, Finland; 5 Department of Knowledge Brokers, Finnish Institute for Health and Welfare, Helsinki, Finland; 6 Region Stockholm, Academic Primary Health Care Centre, Stockholm, Sweden; 7 Department of Molecular Medicine and Surgery, Karolinska Institutet, Stockholm, Sweden; 8 Clinical Medicine Research Unit, Medical Research Center Oulu, Oulu University Hospital and University of Oulu, Oulu Finland; 9 Department of Clinical and Molecular Medicine, Norwegian University of Science and Technology, Trondheim, Norway; 10 Stem Cells and Metabolism Research Program, Faculty of Medicine, University of Helsinki, Helsinki, Finland; 11 Department of Paediatrics, Labatt Family Heart Centre, The Hospital for Sick Children, University of Toronto, Toronto, Ontario, Canada

## Abstract

**Importance:**

Maternal diabetes and overweight or obesity are known to be associated with increased risk of congenital heart defects (CHDs) in offspring, but there are no large studies analyzing outcomes associated with these factors in 1 model.

**Objective:**

To investigate the association of maternal diabetes and overweight or obesity with CHDs among offspring in 1 model.

**Design, Setting, and Participants:**

This nationwide, population-based register study was conducted in a birth cohort from Finland consisting of all children born between 2006 and 2016 (620 751 individuals) and their mothers. Data were analyzed from January 2022 until November 2023.

**Exposures:**

Maternal prepregnancy body mass index (BMI; calculated as weight in kilograms divided by height in meters squared), categorized as underweight (<18.5), normal (18.5-24.9), overweight (25.0-29.9), and obesity (≥30), was assessed. Maternal diabetes status, classified as no diabetes, type 1 diabetes (T1D), type 2 or other diabetes, and gestational diabetes, was assessed.

**Main Outcomes and Measures:**

Odds ratios (ORs) of isolated CHDs in children were found. In addition, 9 anatomical CHD subgroups were studied.

**Results:**

Of 620 751 children (316 802 males [51.0%]; 573 259 mothers aged 20-40 years [92.3%]) born in Finland during the study period, 10 254 children (1.7%) had an isolated CHD. Maternal T1D was associated with increased odds of having a child with any CHD (OR, 3.77 [95% CI, 3.26-4.36]) and 6 of 9 CHD subgroups (OR range, 3.28 [95% CI, 1.55-6.95] for other septal defects to 7.39 [95% CI, 3.00-18.21] for transposition of great arteries) compared with no maternal diabetes. Maternal overweight was associated with left ventricular outflow tract obstruction (OR, 1.28 [95% CI, 1.10-1.49]) and ventricular septal defects (OR, 0.92 [95% CI, 0.86-0.98]), and obesity was associated with complex defects (OR, 2.70 [95% CI, 1.14-6.43]) and right outflow tract obstruction (OR, 1.31 [95% CI, 1.09-1.58]) compared with normal maternal BMI.

**Conclusions and Relevance:**

This study found that maternal T1D was associated with increased risk for most types of CHD in offspring, while obesity and overweight were associated with increased risk for complex defects and outflow tract obstruction and decreased risk for ventricular septal defects. These different risk profiles of T1D and overweight and obesity may suggest distinct underlying teratogenic mechanisms.

## Key Points

**Question:**

Is there an association between maternal diabetes and overweight and offspring congenital heart defects (CHDs)?

**Findings:**

In this cohort study of 620 751 children, maternal type 1 diabetes (T1D) was associated with a 3.77-fold increase in adjusted odds for any CHD in offspring, and the risk was increased in 6 of 9 CHD subgroups. There were associations of overweight and obesity with odds of offspring CHD in few anatomical subgroups.

**Meaning:**

This study found that T1D was a risk factor associated with nearly all subtypes of CHD in offspring, while overweight and obesity were associated with certain types of CHD, suggesting distinct teratogenic mechanisms.

## Teaser

This cohort study investigates the association of maternal diabetes and body mass index with congenital heart defects in offspring.

## Introduction

Congenital heart defects (CHDs) are the most common congenital malformations in children, traditionally thought to occur in approximately 1 in 100 newborns.^1^ Together with prematurity and birth asphyxia and trauma, CHDs are among the leading causes of deaths in the first year of life in high-resource settings.^2^ Although most children with CHDs survive to adulthood, CHDs are associated with significant mortality, morbidity, and reduced quality of life.^3,4,5^ It has been estimated that nearly 12 million people were living with CHDs globally in 2017.^2^

While there clearly is a hereditary component associated with CHD, potentially modifiable maternal factors, such as maternal overweight, obesity, and pregestational and gestational diabetes (PGD and GD) have been associated with increased risk for CHD in offspring.^6,7,8,9,10^ The role of maternal type 1 diabetes (T1D) as a significant risk factor has been well documented, but the significance of GD and maternal obesity and overweight is less clear, especially for specific CHD subgroups. Moreover, to our knowledge, there are no large studies investigating maternal diabetes and obesity in the same model. Given that these conditions often occur in parallel, better understanding of the contribution of each factor to offspring risk for CHD in general and for specific CHD subgroups may not only aid prevention, but also provide cues to direct future research in unraveling underlying molecular-level mechanisms. We investigated the association of maternal PGD, GD, and overweight and obesity with the risk of isolated CHDs and CHDs of selected subgroups in a nationwide register study from Finland.

## Methods

This cohort study was approved by the Research Ethics board of the Finnish Institute for Health and Welfare and relevant register authorities. No informed consent from registered persons is required for the use of pseudonymized register data for research purposes in Finland. The reporting of the study followed the Strengthening the Reporting of Observational Studies in Epidemiology (STROBE) reporting guideline.

### Study Design and Setting

We conducted a nationwide register study in Finland including all children born (liveborn and stillborn) in 2006 to 2016 and their mothers (eFigure 1 in Supplement 1). Exclusion criteria included missing information on gestational age, unclear CHD diagnosis, and diagnosis for syndromes, chromosomal aberrations, or extracardiac malformations (major anomalies according to EUROCAT)^11^ (eFigure 1 and eTable 1 in Supplement 1).

### Data Sources

Data were collected from the following national registers: the Medical Birth Register (MBR), Register of Congenital Malformations (RCM), and Care Register for Health Care (CRHC) maintained by the Finnish Institute for Health and Welfare and the Register of Special Reimbursements for Prescription Medicines maintained by the Social Insurance Institution of Finland.^12,13^ Every Finnish citizen and permanent resident has a personal identification number, which enables linkage of information between registers. Information on maternal and paternal education was received from Statistics Finland. A detailed description of these registers is provided in the eMethods in Supplement 1.

### Outcomes

The main outcome was isolated CHD in the child obtained from the RCM. Isolated CHD was defined as having a diagnosis for 1 or more CHDs and not having diagnoses for chromosomal aberrations, syndromes, or any other major extracardiac anomaly (eTable 1 in Supplement 1). Isolated CHDs were divided into 9 groups according to their anatomical origin: atrial septal defects, ventricular septal defects (VSD), other septal defects, transposition of great arteries, left ventricular outflow tract obstruction (LVOTO), right ventricular outflow tract obstruction (RVOTO), pulmonary venous anomalies, anomalies of thoracic arteries and veins, and complex defects (eTable 2 in Supplement 1). The complex group included rare severe diagnoses, such as isomerisms, double outlet left ventricle, double inlet left ventricle, and some complex combined defects.

### Exposures and Covariates

Maternal body mass index (BMI; calculated as weight in kilograms divided by height in meters squared) was calculated from the weight and height reported in the MBR. Height was self-reported. Prepregnancy weight is self-reported during the first antenatal clinic visit, when weight is also measured. BMI was categorized as underweight (<18.5), normal (18.5-24.9), overweight (25.0-29.9), and obese (≥30.0). Maternal diabetes was classified as no diabetes, T1D, type 2 or other diabetes (T2D), and GD according to information in the MBR, CRHC, and Register of Special Reimbursements for Prescription Medicines (eMethods and eTable 3 in Supplement 1).

Covariates considered as potential confounders included year of birth, maternal age, parity, maternal smoking during pregnancy (yes or no), and highest parental education level, obtained from MBR and Statistics Finland. Data on highest parental completed education during the study period were categorized according to the International Standard Classification of Education 2011 (ISCED) as low (ISCED classes 0-2), intermediate (ISCED classes 3-5), high (ISCED classes 6-8), or missing.^14^

### Statistical Analysis

Categorical variables were reported as frequencies and percentages and continuous variables as means and SDs. First, logistic regression adjusted for child birth year was used for analyzing the association of maternal BMI classes with isolated CHDs and selected CHD subgroups in offspring. Similarly, logistic regression analysis adjusted for child birth year was used for analyzing the association of maternal diabetes with isolated CHDs and the same CHD subgroups.

Then, multivariable logistic regression analysis was used to analyze the association of maternal diabetes and BMI with isolated CHDs and selected CHD subgroups in offspring, and the model was adjusted for the previously mentioned covariates. Diabetes and BMI were assessed in the same analysis given that the conditions often occur in parallel. Finally, multiplicative interaction of maternal diabetes and BMI with isolated CHDs and CHD subgroups was analyzed using logistic regression analysis, first by adjusting for child birth year and then adjusted for the previously mentioned covariates.

Results of logistic regression are presented as odds ratios (ORs) with 95% CIs as measures of associations. Individuals with missing data were excluded from the multivariable logistic regression analysis. The comparison of isolated CHD frequencies between individuals with missing and those with nonmissing data was made with the χ^2^ test.

Population-attributable risk was calculated using an indirect method standardized by maternal age to analyze the risk for isolated CHDs in members of the total population that is attributable to risk factors, which were maternal diabetes and obesity. The attributable risk was calculated to analyze the proportion of isolated CHDs in children exposed to maternal diabetes or obesity.

Analyses were performed in January 2022 until November 2023. We considered 2-sided *P* values less than .05 statistically significant. Data handling and analyses were performed using SAS Enterprise Guide statistical software versions 7.1 and 8.3 (SAS Institute).

## Results

The study population consisted of 620 751 children (316 802 males [51.0%]; 573 259 mothers aged 20-40 years [92.3%]), of whom 10 254 children (1.7%) had an isolated CHD and 610 497 children did not have CHDs (98.3%) (eTable 4 in Supplement 1). There were 4932 males (48.1%) with CHDs and 311 870 males (51.1%) without CHDs. Baseline characteristics of individuals in the study and maternal pregnancy data are presented in Table 1.

**Table 1.** Baseline Characteristics of Study Population

Characteristic	Children, No. (%) (N = 620 751)^a^	
	Isolated CHD (n = 10 254)	Control group (n = 610 497)
Sex		
Male	4932 (48.1)	311 870 (51.1)
Female	5322 (51.9)	298 596 (48.9)
Highest parental education level^b^		
1 (Low)	283 (2.8)	17 460 (2.9)
2 (Intermediate)	4648 (45.3)	269 594 (44.2)
3 (High)	4852 (47.3)	294 378 (48.2)
**Maternal characteristics**		
Age, y		
Mean (SD)	29.9 (5.4)	29.8 (5.3)
≤20	392 (3.8)	24 405 (4.0)
21-39	9465 (92.3)	563 794 (92.4)
≥40	397 (3.9)	22 298 (3.7)
Smoking	1630 (15.9)	91 518 (15.0)
Parity, mean (SD)	1.1 (1.4)	1.1 (1.4)
Diabetes		
T1D	228 (2.2)	3466 (0.6)
T2D or other	33 (0.3)	1068 (0.2)
GD	1524 (14.9)	85 503 (14.0)
No diabetes	8469 (82.6)	520 460 (85.3)
BMI		
<18.5	393 (3.8)	21 898 (3.6)
18.5-24.9	6192 (60.4)	372 584 (61.0)
25-29.9	2177 (21.2)	130 008 (21.3)
≥30	1298 (12.7)	73 747 (12.1)
Any diabetes and obesity^c^		
Diabetes+ and obesity+	1109 (10.8)	57 901 (9.5)
Diabetes+ and obesity−	655 (6.4)	30 685 (5.0)
Diabetes− and obesity+	2366 (23.1)	145 854 (23.9)
Diabetes− and obesity−	5930 (57.8)	363 797 (59.6)

Abbreviations: BMI, body mass index (calculated as weight in kilograms divided by height in meters squared); CHD, congenital heart defect; GD, gestational diabetes; T1D, type 1 diabetes; T2D, type 2 diabetes.

^a^ Data were missing for maternal smoking status in 14 845 individuals (2.4%), maternal BMI in 12 454 individuals (2.0%), and highest parental education level in 29 536 individuals (4.8%).

^b^ The highest education level of either parent is indicated, and the level of education is categorized as level 1, level 2, and level 3, indicating low, intermediate, and high education levels.

^c^ Diabetes+ indicates mothers with T1D, T2D, or GD. Obesity+ indicates mothers with a BMI of 25 or greater. Diabetes− indicates mothers without T1D, T2D, or GD. Obesity− indicates mothers with a BMI less than 25.

The prevalence of GD increased during the study period, from 6050 of 58 545 mothers in 2006 (10.3%) to 10 196 of 53 087 mothers in 2016 (19.2%), whereas the prevalence of T2D increased from 79 mothers (0.1%) to 165 mothers (0.3%) and the prevalence of T1D was stable at approximately 0.7% (338 mothers in 2006 [0.6%] and 343 mothers in 2016 [0.7%]) (eFigure 2 in Supplement 1). Maternal overweight increased from 11 860 mothers (20.3%) to 11 797 mothers (22.2%) and maternal obesity from 6283 mothers (10.7%) to 7097 mothers (13.3%) (eFigure 3 in Supplement 1). GD occurred more frequently as BMI increased in mothers of children with CHDs, as 505 of 6192 mothers with normal BMI (8.2%), 459 of 2177 mothers with overweight (21.1%), and 534 of 1298 mothers with obesity (41.1%) had a GD diagnosis; similar results were observed in mothers of children without CHDs (eTable 5 in Supplement 1).

**Figure 1.**
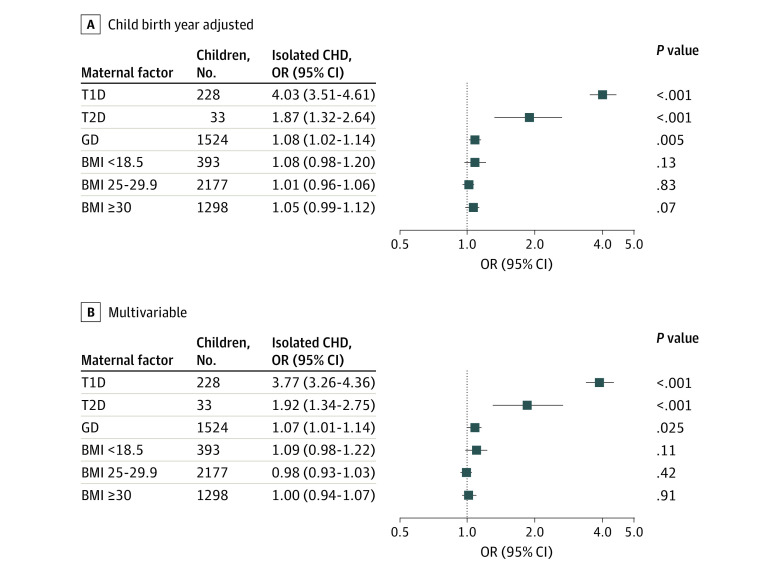
Association Between Maternal Factors and Isolated Congenital Heart Defects (CHDs) The association of maternal body mas index (BMI; calculated as weight in kilograms divided by height in meters squared) and diabetes with CHDs was investigated using child birth year–adjusted (A) and multivariable (B) logistic regression analysis. Multivariable analysis was adjusted to maternal smoking, maternal age, child birth year, first parity, and highest parental education level. Normal BMI (18.5-24.9) and no diabetes were used as reference groups in logistic regression analysis. Statistical significance was reached with *P* < .05. GD indicates gestational diabetes; OR, odds ratio; T1D, type 1 diabetes; T2D, type 2 diabetes.

In logistic regression adjusted for child birth year, maternal T1D (OR, 4.03 [95% CI, 3.51-4.61]), T2D (OR, 1.87 [95% CI, 1.32-2.64]), and GD (OR, 1.08 [95% CI, 1.02-1.14]) were associated with increased odds of isolated CHDs in offspring compared with no maternal diabetes, while there were no associations of maternal overweight (OR 1.01 [95% CI 0.96–1.06]) or obesity (OR, 1.05 [95% CI, 0.99-1.12]) with isolated CHDs in offspring compared with normal maternal BMI (Figure 1A). In a multivariable logistic regression analysis, maternal T1D (OR, 3.77 [95% CI, 3.26-4.36]) and T2D (OR, 1.92 [95% CI, 1.34-2.75]) remained associated with increased odds of CHDs compared with no maternal diabetes (Figure 1B). We found no association between maternal obesity (OR, 1.00 [95% CI, 0.94-1.07]) or overweight (OR, 0.98 [95% CI, 0.93-1.03]) and isolated CHDs in offspring in the multivariable logistic regression analysis when CHDs were analyzed as a single group. There was no multiplicative interaction of maternal diabetes and BMI with isolated CHDs in logistic regression adjusted for child birth year or in multivariable logistic regression analysis.

### CHD Subgroup Analyses

**Figure 2.**
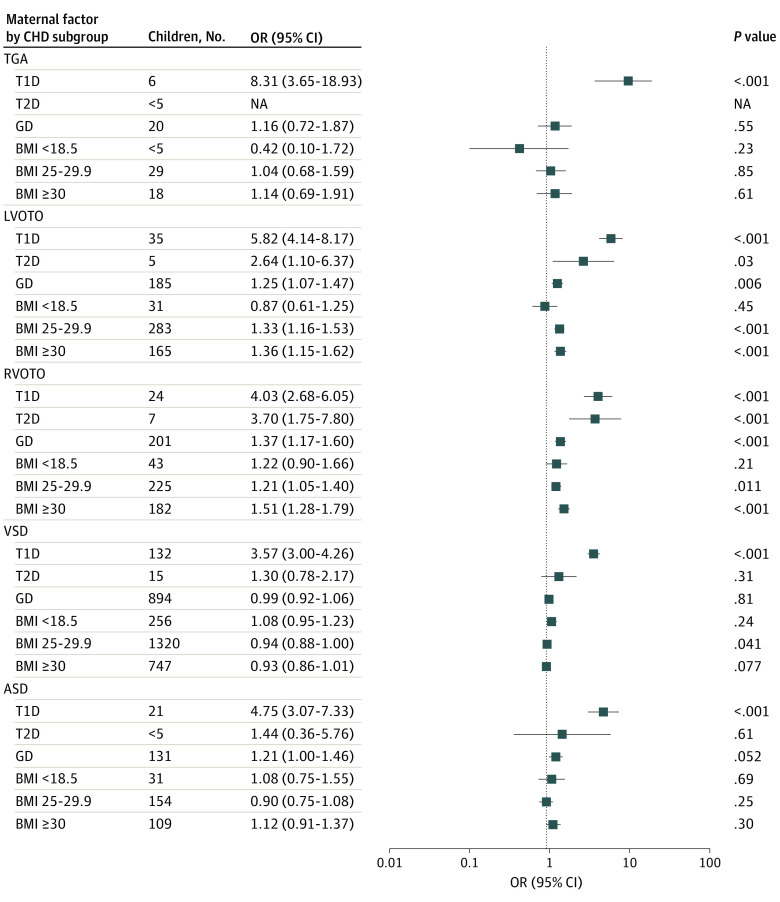
Association Between Maternal Factors and Congenital Heart Defect (CHD) Subgroups The analysis of the association of maternal body mass index (BMI; calculated as weight in kilograms divided by height in meters squared) and diabetes with offspring CHD subgroup was adjusted by birth year of the child. Normal BMI (18.5-24.9) and no diabetes were used as reference groups. Statistical significance was reached with *P* <. 05. Factors with NA could not be analyzed owing to low population numbers. ASD indicates atrium septal defect; GD, gestational diabetes; LVOTO, left ventricle outflow tract obstruction; NA, not applicable; OR, odds ratio; RVOTO, right ventricle outflow tract obstruction; T1D, type 1 diabetes; T2D, type 2 diabetes or other diabetes; TGA, transposition of great arteries; VSD, ventricular septal defect.

**Figure 3.**
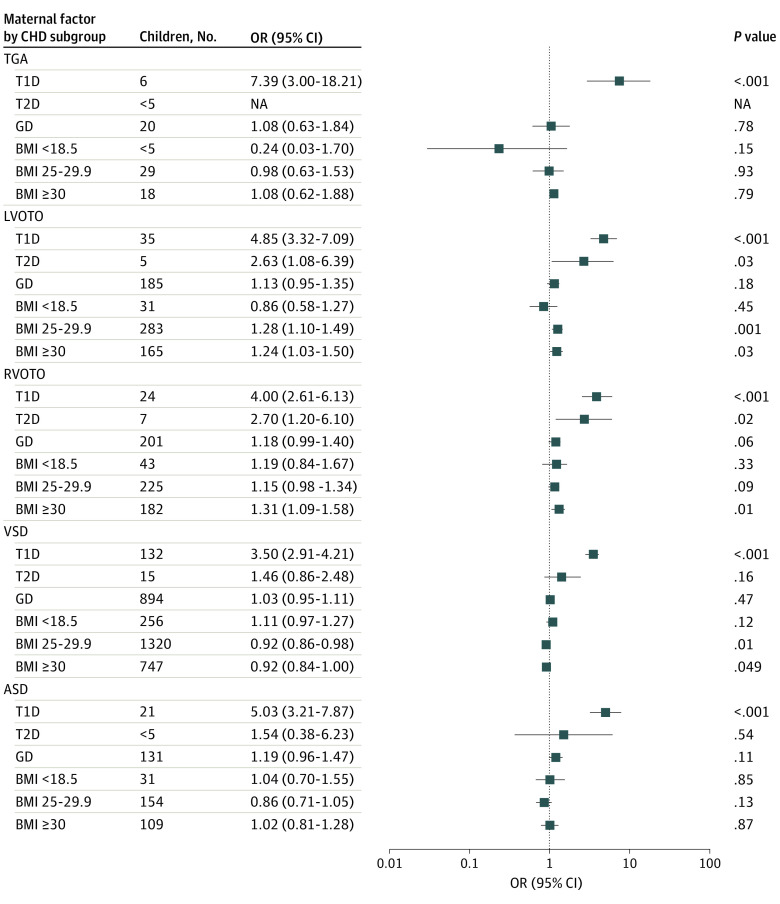
Association Between Maternal Factors and Congenital Heart Disease (CHD) Subgroups in Multivariable Analysis The multivariable logistic regression analysis of the association of maternal body mass index (BMI; calculated as weight in kilograms divided by height in meters squared) and diabetes with CHD subgroups was adjusted to maternal smoking, maternal age, birth year of the child, first parity, and highest parental education level. Normal BMI (18.5-24.9) and no diabetes were used as reference groups. Statistical significance was reached with *P* < .05. Factors with NA could not be analyzed owing to low population numbers. ASD indicates atrium septal defect; GD, gestational diabetes; LVOTO, left ventricle outflow tract obstruction; NA, not applicable; OR, odds ratio; RVOTO, right ventricle outflow tract obstruction; T1D, type 1 diabetes; T2D, type 2 diabetes or other diabetes; TGA, transposition of great arteries; VSD, ventricular septal defect.

The logistic regression analysis with offspring CHD subgroups as outcomes and adjusted for birth year is presented in Figure 2 and eFigure 4 in Supplement 1. After models were adjusted for other covariates (Figure 3; eFigure 5 in Supplement 1), T1D was associated with the greatest increase in risk for transposition of great arteries (OR, 7.39 [95% CI, 3.00-18.21]), LVOTO (OR, 4.85 [95% CI, 3.32-7.09]), RVOTO (OR, 4.00 [95% CI, 2.61-6.13]), isolated atrial septal defect (OR, 5.03 [95% CI, 3.21-7.87]), isolated VSD (OR, 3.50 [95% CI, 2.91-4.21]), and other septal defects (OR, 3.28 [95% CI 1.55-6.95]) compared with no maternal diabetes. Maternal overweight was associated with increased odds of LVOTO (OR, 1.28 [95% CI, 1.10-1.49]) compared with normal maternal BMI. Maternal obesity was associated with increased odds of complex defects (OR, 2.70 [95% CI 1.14-6.43]), LVOTO (OR, 1.24 [95% CI, 1.03-1.50]), and RVOTO (OR, 1.31 [95% CI, 1.09-1.58]) compared with normal maternal BMI. Finally, maternal overweight was associated with lower odds of VSD (OR, 0.92 [95% CI, 0.86-0.98]) in offspring and maternal underweight was associated with increased odds of pulmonary venous anomalies (OR, 6.75 [95% CI, 2.43-18.77]) compared with normal maternal BMI.

There was a multiplicative interaction between maternal diabetes and BMI (mothers with overweight compared with mothers with normal BMI) in the association with LVOTO in logistic regression adjusted for child birth year (OR, 0.66 [95% CI, 0.48-0.89]; *P* = .03) but not in multivariable logistic regression analysis. Otherwise, there were no interactions between maternal diabetes and BMI in associations with other CHD subgroups.

### Attributable and Population-Attributable Risk

We determined the attributable risk and population-attributable risk with overweight or obesity and diabetes associated with CHD in offspring (Table 2). The attributable risk of diabetes was 17.20% (95% CI, 13.20% to 20.90%); that is, 17.20% of the risk of having offspring with CHDs in the maternal diabetes group was attributed to any maternal diabetes. The population-attributable risk of any diabetes was 3.00% (95% CI, 2.20% to 3.80%); that is, 3% of the risk of having offspring with CHDs in the whole population was attributed to any maternal diabetes. There was no significant attributable risk (1.87% [95% CI −0.05% to 1.78%) or population-attributable risk (0.65% [95% CI −0.50% to 1.78%) for maternal overweight and obesity.

**Table 2.** Risk of Isolated CHD by Maternal Risk Factor

Risk factor	Isolated CHD	
	PAR (95% CI), %	Attributable risk (95% CI), %
Any diabetes^a^	3.00 (2.20 to 3.80)	17.2 (13.2 to 20.9)
BMI ≥25	0.65 (−0.50 to 1.78)	1.87 (−0.05 to 1.78)
T1D	1.93 (1.59 to 2.27)	73.6 (70.0 to 76.6)
T2D	0.18 (0.04 to 0.31)	45.2 (17.4 to 59.0)
GD	1.23 (0.47 to 2.00)	8.07 (3.26 to 12.4)

Abbreviations: BMI, body mass index (calculated as weight in kilograms divided by height in meters squared); CHD, congenital heart defect; GD, gestational diabetes; PAR, population-attributable risk; T1D, type 1 diabetes; T2D, type 2 diabetes.

^a^ Any diabetes includes GD, T1D, and T2D.

### Missing Data

Data were missing on maternal smoking status during pregnancy in 14 845 individuals (2.4%), maternal BMI in 12 454 individuals (2.0%), and highest parental education level in 29 536 individuals (4.8%). There were no differences in outcomes for individuals with missing and nonmissing data in the study population (eTable 6 in Supplement 1).

## Discussion

This nationwide register cohort study quantified the increase in risk of offspring CHDs that was associated with maternal diabetes and overweight or obesity in pregnancy. While maternal T1D was associated with the greatest increase in risk, with 3.77-fold increased odds for any CHD and an association with increased risk in most CHD subcategories, maternal overweight and obesity were associated with increased risk for only complex defects and outflow tract obstruction defects. Intriguingly, maternal overweight was associated with lower odds of VSD in offspring. Furthermore, there was no interaction of maternal diabetes and BMI in the association with isolated CHD, suggesting that both were individual risk factors associated with the outcome. These results may suggest that maternal diabetes and overweight or obesity have distinct teratogenic mechanisms given that associated changes in odds were different for many CHD subgroups, and in some cases even opposite.

In general, our study indicated that maternal overweight and obesity were associated with smaller increases in CHD odds in offspring than previously reported. We speculate that this may be due to our comprehensive data on maternal diabetes, which likely accounts for a larger part of the risk in individuals with overweight and obesity than previously thought. This is in line with a recent smaller study from China^15^ showing that PGD partially mediated the association between maternal obesity and CHD. In our study, maternal overweight and obesity were associated with an increased risk for complex defects and outflow tract obstruction defects, whereas 2 large previous studies found associations with a wider range of defects and with higher ORs.^6,8^ These studies, however, did not adjust for maternal diabetes comprehensively. The study by Persson et al^8^ on 28 628 individuals with CHDs and 2 050 491 individuals without CHDs excluded those with PGD from the analysis and lacked GD as a covariate in the multivariate model. The study by Madsen et al,^6^ including 11 263 individuals with CHDs and 140 470 individuals in the control group, presented no information on T1D. Although the authors adjusted analyses for GD, the prevalence of GD was 6.3% in mothers of offspring with CHDs and 4.3% in those in the control group, and thus GD was potentially underreported. In our study, GD occurred frequently with BMI in mothers of children with CHDs. However, there was no interaction between maternal diabetes and BMI in the association with isolated CHDs in general, and a small interaction association was observed for LVOTO defects. Thus, it is likely that previous studies underestimated the role of maternal diabetes in their analyses.

In several smaller studies showing no association between maternal BMI and offspring CHDs or an association with some increase in risk of CHDs, findings on the association with specific CHD subtypes were inconsistent.^7,16,17,18,19^ Indeed, analyses of CHD subtypes were limited by low prevalence of individual malformations, even in large cohorts. A 2019 meta-analysis^20^ of 19 studies with 2 416 546 participants reported pooled relative risks of having infants with CHDs of 1.08 (95% CI, 1.03-1.13) in mothers with overweight and 1.23 (95% CI, 1.17-1.29) in mothers with obesity. Nonetheless, most included studies were case-control studies, and few adjusted for confounding factors, such as maternal age, smoking, education, or diabetes. Moreover, many studies used BMI based on retrospective, self-reported data, which are subject to recall bias.

Interestingly, our results indicated a lower risk for VSD in offspring of mothers with overweight, while previous reports have shown no association^8^ or an increased risk.^7^ Maternal obesity has been shown to be associated with increased birth weight in offspring^21,22^ and increased left ventricle^23^ and interventricular septum^24^ thickness during infancy. Thus, one explanation could be that increased septum thickness in these infants may contribute to the closure of small muscular defects before they are diagnosed. However, maternal T1D is also known to be associated with ventricular hypertrophy in offspring in the neonatal period,^25^ but unlike maternal overweight, it is also associated with an increased risk for offspring VSD, as shown by us and others. This suggests that whereas T1D is associated with abnormal cardiac septation, maternal overweight and obesity are not, pointing to different teratogenic mechanisms in these 2 conditions. However, it should be noted that the true prevalence of isolated VSD is difficult to estimate, which could have had an impact on the specific assessment of the association of investigated risk factors with isolated VSD.^26,27^

The association of maternal T1D with offspring risk for CHDs is well known. In line with previous studies,^9,28,29,30^ our results demonstrated a 3- to 4-fold increased risk for any CHD, and the risk was increased for all CHD subgroups that had enough individuals to determine it. GD was associated with increased risk for any CHD and LVOTO defects in the logistic regression adjusted for child birth year; however, there were no associations for these outcomes in the multivariable analysis. Given that GD is highly prevalent, occurring in 1 in 3 expectant mothers, any risk increase is important at the population level. This was demonstrated by a population-attributable risk of 1.23% for GD compared with 1.93% for T1D, which is considerably less prevalent.

During the 2006 to 2016 study period, the screening policy of GD in Finland was changed from risk factor based to comprehensive screening, introduced by a 2008 guideline. This resulted in an increased number of women screened and milder cases included as GD with less severe mean perinatal and neonatal outcomes.^31,32^ In addition, the proportion of parturient individuals who were obese and older (aged ≥35 years) increased during the study period,^33^ which may also be associated with the increase in GD prevalence. These changes were also likely to affect the association found between diagnosed GD and CHDs.

Higher plasma glucose values during early pregnancy have been associated with an increased risk for CHDs in offspring in mothers without diabetes,^34^ and hyperglycemia is likely the major teratogenic factor in T1D.^35,36^ The pathophysiological processes behind GD and obesity-associated risk are less well known. It is not unreasonable to speculate that at least some mothers diagnosed with GD later in pregnancy have glycemic dysregulation and pathologically high glucose values already in early pregnancy; however, additional mechanisms should be considered. Studies from 2015 to 2020^37,38,39^ have demonstrated distinct early pregnancy metabolomic profiles, including exaggerated dyslipidemia and increased inflammatory markers in mothers who later developed GD. Abnormal early pregnancy maternal lipid profiles have been associated with increased risk for CHDs in offspring.^40,41^ Obesity and GD are associated with increased oxidative stress and endothelial dysfunction, and endocardial dysfunction has been proposed as 1 pathophysiological origin of LVOTO defects.^42,43,44^ These findings suggest that obesity and GD-mediated abnormal metabolomics related to inflammation, oxidative stress, and hyperlipidemia could be associated with an additive risk in individuals who are genetically predisposed. The association of maternal overweight and obesity with risk of offspring LVOTO, RVOTO, and complex defects warrants further mechanistic research on these CHD subtypes to identify potentially modifiable pathophysiological processes.

The strengths of this study include using nationwide register data with an unselected population of all children born in 2006 to 2016 in Finland. The data on exposures and outcomes were prospectively collected and comprehensively and reliably defined. Maternal diabetes was defined based on 3 unrelated registers, and we assessed associations of PGD and GD separately. In addition, we were able to assess the severity of obesity. For the analysis, we included only individuals with isolated heart defects without syndromes or any other major malformations, leading to exclusion of most individuals with definitive or probable larger structural genetic defects with likely different etiologies.

### Limitations

This study has several limitations. To our knowledge, it is thus far the largest population-based study to address the combined association of diabetes and obesity with offspring risk for CHDs. Although we used a national, 11-year cohort, the number of individuals in CHD subgroups remained limited, which was seen as large CIs in many significant findings. Another important limitation was that the data on pregnancy terminations and miscarriages were not reliably available. Congenital anomalies are known to be common in miscarriages, and approximately 350 pregnancy terminations are made annually due to major congenital anomalies in Finland. It is likely that some associations were missed because we did not have these data, and future studies including pregnancy terminations and stillbirths before 22 gestational weeks would be important. Additionally, health care database data (particularly self-reported measures, such as height and weight used for determining BMI) are not always 100% accurate, and it is possible that these led to some inaccuracies in results.

## Conclusions

This cohort study emphasizes T1D as a risk factor associated with offspring CHDs, whereas GD and maternal overweight and obesity were associated with a smaller increase in risk, at least in this high-resource setting with universal antenatal care. However, with increasing prevalence of GD and maternal overweight, the risk at the population level is substantial. It has been shown that standard treatment of maternal diabetes is associated with reduced risk of anatomical malformations in offspring.^45^ Thus, primary prevention of maternal overweight and obesity and careful treatment of PGD may hold the opportunity to reduce the burden of disease. Finally, a better understanding of the underlying mechanisms of maternal overweight and obesity in increased offspring risk for LVOTO, RVOTO, and complex defects may further improve the prevention of these CHD subtypes.

## Notes

Supplement 1.
eMethods.
**eTable 1.** Diagnoses Used in Excluding Individuals With Extracardiac Anomalies **eTable 2.** Congenital Heart Defect Subgroup Categories Were Classified According to Atlanta ICD-9 Classification **eTable 3.** ICD-9 Codes and Special Reimbursement Codes Used in Definition of Maternal Diabetes From Registries **eTable 4.** Prevalence of Congenital Heart Defects in Study Population **eTable 5.** Presence of Maternal Gestational Diabetes and No Diabetes by Body Mass Index in Mothers of Children With and Without Isolated Congenital Heart Defects **eTable 6.** Comparison Between Individuals With Missing and Nonmissing Data and Isolated Congenital Heart Defects **eFigure 1.** Flowchart of Selection of Study Participants **eFigure 2.** Prevalence of Maternal Diabetes During Study Period **eFigure 3.** Prevalence of Maternal Obesity and Congenital Heart Defects During Study Period **eFigure 4.** Association of Maternal Body Mass Index and Diabetes With All Congenital Heart Defect Subgroups **eFigure 5.** Association of Maternal Body Mass Index and Diabetes With All Congenital Heart Defect Subgroups Using Multivariable Logistic Regression Analysis

Supplement 2.
Data Sharing Statement
