# Supplementary material for: Maternal Diabetes and Overweight and Congenital Heart Defects in Offspring
Source: JAMA Netw Open. 2024 Jan 5;7(1):e2350579. doi: 10.1001/jamanetworkopen.2023.50579 (PMC10770771; doi:10.1001/jamanetworkopen.2023.50579)
Supplement: Supplement 1. — eMethods. eTable 1. Diagnoses Used in Excluding Individuals With Extracardiac Anomalies eTable 2. Congenital Heart Defect Subgroup Categories Were Classified According to Atlanta ICD-9 Classification eTable 3. ICD-9 Codes and Special Reimbursement Codes Used in Definition of Maternal Diabetes From Registries eTable 4. Prevalence of Congenital Heart Defects in Study Population eTable 5. Presence of Maternal Gestational Diabetes and No Diabetes by Body Mass Index in Mothers of Children With and Without Isolated Congenital Heart Defects eTable 6. Comparison Between Individuals With Missing and Nonmissing Data and Isolated Congenital Heart Defects eFigure 1. Flowchart of Selection of Study Participants eFigure 2. Prevalence of Maternal Diabetes During Study Period eFigure 3. Prevalence of Maternal Obesity and Congenital Heart Defects During Study Period eFigure 4. Association of Maternal Body Mass Index and Diabetes With All Congenital Heart Defect Subgroups eFigure 5. Association of Maternal Body Mass Index and Diabetes With All Congenital Heart Defect Subgroups Using Multivariable Logistic Regression Analysis [file jamanetwopen-e2350579-s001.pdf]

Supplemental Online Content

Turunen R, Pulakka A, Metsälä J, et al. Maternal diabetes and overweight as risk factors associated with congenital heart defects in offspring. *JAMA Netw Open*. 2024;7(1):e2350579. doi:10.1001/jamanetworkopen.2023.50579

eMethods.
eTable 1. Diagnoses Used in Excluding Individuals With Extracardiac Anomalies
eTable 2. Congenital Heart Defect Subgroup Categories Were Classified According to Atlanta ICD-9 Classification
eTable 3. ICD-9 Codes and Special Reimbursement Codes Used in Definition of Maternal Diabetes From Registries
eTable 4. Prevalence of Congenital Heart Defects in Study Population
eTable 5. Presence of Maternal Gestational Diabetes and No Diabetes by Body Mass Index in Mothers of Children With and Without Isolated Congenital Heart Defects
eTable 6. Comparison Between Individuals With Missing and Nonmissing Data and Isolated Congenital Heart Defects
eFigure 1. Flowchart of Selection of Study Participants
eFigure 2. Prevalence of Maternal Diabetes During Study Period
eFigure 3. Prevalence of Maternal Obesity and Congenital Heart Defects During Study Period
eFigure 4. Association of Maternal Body Mass Index and Diabetes With All Congenital Heart Defect Subgroups
eFigure 5. Association of Maternal Body Mass Index and Diabetes With All Congenital Heart Defect Subgroups Using Multivariable Logistic Regression Analysis

This supplemental material has been provided by the authors to give readers additional information about their work.

© 2024 Turunen R et al. *JAMA Network Open*.

eMethods

Detailed description of used registers

The MBR includes data on live births and on stillbirths of fetuses with a birth weight of at least 500 g or with a gestational age of at least 22 weeks. These data are collected using standardized forms and the register includes information on maternal medical history, pregnancy, and delivery and infant's early medical history. The RCM contains data on congenital chromosomal and structural anomalies that have been detected or suspected in stillborn and live born infants and fetuses. Major congenital anomalies are defined according to EUROCAT classification[1]. The register includes only cases with at least one major anomaly. The RCM uses Atlanta ICD-9 codes for diagnosis and descriptive diagnosis of congenital anomalies. The CRHC contains in- and outpatient visit data, including diagnoses, in hospitals and health centers.

The data on special reimbursements for prescription medicines are based on the reimbursement of outpatient pharmaceutical expenses. All permanent residents of Finland are covered under the Finnish National Health Insurance system and are eligible for reimbursements for the cost of medicines prescribed by a doctor or a dentist. Entitlement for special medication reimbursement is granted based on a clinician's statement, reviewed by a physician at SII against defined criteria. Special reimbursement is designated with a reimbursement code indicating the medication category (e.g. antidiabetic medication) and an ICD-10 code since 2000. Special reimbursement is not granted for gestational diabetes.

Definition of diabetes

Diabetes types were defined stepwise starting with T1DM that ruled out T2DM and GDM. After defining T1DM, T2DM was defined by ruling out GDM. T1DM and T2DM were classified by the respective diagnosis codes in the granted special reimbursement for the costs of insulin purchases or in the CRHC (eTable 3). GDM was defined as previously described using the information in the MBR (pathological oral glucose tolerance test (OGTT), insulin started during pregnancy, and correct diagnosis codes)[2].

During the 2006–2016 study period the screening policy of GDM was changed from risk factor based to comprehensive screening in 2008. Current Care Guidelines recommended a 75 g two-hour oral glucose tolerance test at weeks 24-28 for all women except those with very low GDM risk. For women with high risk, an additional oral glucose tolerance test was recommended at 12-16 weeks. The 2008 guideline, which was gradually implemented over the following years, also unified the cut-off concentrations for venous plasma glucose indicating GDM and referred as pathological OGTT to ≥5.3 mmol/l at baseline (fasting glucose), ≥10.0 mmol/l at 1 hour after glucose intake or ≥8.6 mmol/l at 2 hours after glucose intake[3].

References

1. Greenlees R, Neville A, Addor MC, et al. Paper 6: EUROCAT member registries: Organization and activities. *Birth Defects Research Part A: Clinical and Molecular Teratology*. 2011;91(S1):S51-S100. doi:10.1002/bdra.20775

2. Pukkila J, Mustaniemi S, Lingaiah S, et al. Increased Oral Care Needs and Third Molar Symptoms in Women with Gestational Diabetes Mellitus: A Finnish Gestational Diabetes Case-Control Study. *Int J Environ Res Public Health*. 2022;19(17). doi:10.3390/ijerph191710711

3. Keikkala E, Mustaniemi S, Koivunen S, et al. Cohort Profile: The Finnish Gestational Diabetes (FinnGeDi) Study. *Int J Epidemiol*. 2020;49(3):762-763g.

© 2024 Turunen R et al. *JAMA Network Open*.

eTable 1. Diagnoses according to Atlanta ICD-9 codes used in excluding subjects with extracardiac anomalies

Subject groups	Atlanta ICD-9 diagnosis codes
Excluded CHD diagnosis	745410, 745520, 746870, 746887, 747000, 747230, 747260, 747400, 747410, 747430, 759005, 745500
Excluded chromosomal alterations	7580, 7581, 7582, 758000, 758020, 758040, 758010, 758030, 758090, 758008, 758098, 758200, 758220, 758240, 758210, 758230, 758290, 758295, 758208, 758298, 758100, 758120, 758110, 758130, 758190, 758108, 758198, 7585, 758510, 758520, 758530, 758540, 758585,758586, 758580, 758500, 758590, 7583, 758360, 758320, 758310,758300, 758330, 758340, 758350, 758380, 758390, 7584, 758400,758600, 758610, 758690, 748850, 758810, 758800, 758860, 7587, 758700, 758710, 758790, 758840, 758820, 758830, 758890, 758900, 758880, 758990
Excluded syndromes / teratogenic syndromes	742310, 742280, 742480, 742800, 743480, 744880, 759005, 745410, 754010, 753000, 753160, 753180, 755800, 755880, 755810, 756040, 756046, 756110, 756400, 756550, 756410, 756720, 756850, 756800, 757300, 757346, 756045, 757330, 237200, 237700, 759500, 759600, 759610, 759620, 759630, 760700, 760750, 760710, 756030, 756057, 756055, 756060, 756065, 524080, 352600, 759810, 759820, 759800, 759899, 759840, 756830, 759700, 759870, 759860, 759680, 759070, 759340, 279100, 279110, 279910, 758370, 255200, 257800, 742810, 752086, 757520, 759881, 759890, 858610, 59890, 959840, 760718
Extracardiac malformations classified as major anomalies in Registry of Congenital Malformations were excluded to investigate solely isolated congenital heart defects	216902, 228000, 228010, 228100, 228101, 238000, 238010, 238040, 238080, 243990, 425300, 524000, 658800, 740020, 740080, 741, 741000, 741010, 741030, 741050, 741060, 741086, 741087, 741090, 741920, 741930, 741940, 741980, 741985, 741990, 742000, 742080, 742085, 742086, 742090, 7421, 742100, 742200, 742210, 742220, 742230, 742240, 742250, 742260, 742270, 742290, 742300, 742320, 742380, 742385, 742390, 742400, 742410, 742420, 742485, 742490, 742500, 742520, 742530, 742540, 742580, 742880, 742900, 742910, 742990, 743000, 743010, 743100, 743200, 743210, 743300, 743310, 743320, 743326, 743330, 743340, 743380, 743390, 743400, 743410, 743420, 743430, 743440, 743490, 743500, 743510, 743520, 743530, 743535, 743580, 743590, 743600, 743636, 743640, 743660, 743670, 743680, 743690, 7438, 743800, 743900, 744000, 744010, 744020, 744030, 744090, 744110, 7442, 744210, 744280, 744480, 744500, 744800, 744810, 744881, 746002, 746180, 746310, 746680, 746880, 746882, 747325, 747440, 747450,

© 2024 Turunen R et al. *JAMA Network Open*.

Subject groups	Atlanta ICD-9 diagnosis codes
Extracardiac malformations classified as major anomalies in Registry of Congenital Malformations were excluded to investigate solely isolated congenital heart defects	747480, 747490, 7476, 747600, 747610, 747620, 747630, 747640, 747680, 747690, 747800, 747810, 747880, 747900, 748, 748000, 748100, 748120, 748180, 748181, 748208, 748209, 748300, 748310, 748330, 748340, 748350, 748380, 748385, 748390, 748400, 748410, 748480, 748500, 748510, 748520, 748580, 7486, 748620, 748625, 748690, 748810, 748880, 748900, 749, 7490, 749020, 749060, 749070, 749090, 7491, 749100, 749110, 749120, 749170, 749190, 7492, 749200, 749210, 749290, 750110, 750120, 750140, 750180, 7502, 750210, 750230, 750250, 750280, 750300, 750310, 750320, 750330, 750340, 750380, 750420, 750430, 750480, 750580, 750700, 750730, 750750, 750780, 750800, 750900, 750910, 750920, 750990, 751010, 751100, 751110, 751120, 751190, 751200, 751210, 751220, 751230, 751240, 751300, 751310, 751330, 751340, 751400, 751410, 751420, 751490, 751495, 751500, 751520, 751530, 751540, 751550, 751560, 751580, 751590, 751600, 751610, 751620, 751630, 751640, 751650, 751660, 751670, 751700, 751710, 751720, 751730, 751780, 751820, 751880, 7519, 751900, 752000, 752010, 752080, 752085, 752088, 752190, 752200, 752300, 752310, 752380, 752390, 752400, 752410, 752420, 752480, 752490, 752530, 752600, 752605, 752606, 752607, 752610, 752620, 752621, 752625, 752626, 752627, 752700, 752710, 752730, 752790, 7528, 752800, 752820, 752830, 752840, 752850, 752860, 752862, 752865, 752880, 752900, 753001, 753009, 753010, 753100, 753110, 753120, 753130, 753140, 753150, 7532, 753200, 753210, 753220, 753290, 753300, 753310, 753320, 753330, 753380, 753400, 753410, 753420, 753480, 753485, 753490, 753500, 753501, 7536, 753600, 753610, 753620, 753630, 753680, 753690, 7537, 753700, 753710, 753790, 753800, 753820, 753830, 753840, 753850, 753860, 753880, 753900, 753920, 753990, 754001, 754030, 754050, 754061, 754080, 7541, 754100, 754280, 7543, 754300, 754310, 754500, 754580, 754735, 754780, 7548, 754830, 754880, 7550, 755005, 755006, 755007, 755010, 755020,755030, 755090, 755095, 755096, 7551, 755100, 755110, 755120, 755130, 755131, 755190, 755191, 755192, 755193, 755194, 755195, 755196, 755199, 755200, 755210, 755220, 755230, 755240, 755250, 755260, 755270, 755280, 755290, 755300, 755330, 755340, 755350, 755365, 755366, 755380, 755390, 755410, 755480, 755500, 755510, 755520, 755525, 755526, 755530, 755536,755540, 755550,755555, 755556, 755580, 755585, 755610, 755620, 755630, 755631, 755640, 755647, 755650, 755660, 755665, 755666, 755667, 755670, 755680,

© 2024 Turunen R et al. *JAMA Network Open*.

Subject groups	Atlanta ICD-9 diagnosis codes
Extracardiac malformations classified as major anomalies in Registry of Congenital Malformations were excluded to investigate solely isolated congenital heart defects	755685, 755881, 755900, 756005, 756006, 756010, 756020, 756050, 756080, 756081, 756085, 756090, 7561, 756120, 756130, 756140 756145, 756150, 756155, 756156, 756160, 756165, 756166, 756170, 756179, 756180, 756185, 756190, 756300, 756310, 756320, 756330, 756340, 756350, 756380, 756390, 756420, 756430, 756440, 756445, 756446, 756447, 756451, 756460, 756470, 756480, 756490, 756500, 756540, 756560, 756575, 756600, 756610, 756615, 756616, 756617, 756620, 756680, 756700, 756710, 756780, 756790, 756810, 756860, 756880, 756900, 756920, 756990, 757, 757000, 757110, 757115, 757190, 757195, 757196, 757280, 7573, 757320, 757340, 757345, 757350, 757360, 757380, 757382, 757390, 757391, 757395, 757400, 757480, 757500, 7576, 757600, 757620, 757630, 757800, 757990, 758850, 759, 759000, 759010, 759040, 759050, 759080, 759100, 759110, 759180, 759210, 759220, 759240, 759290, 7593, 759300, 759320, 759330, 759390, 7595, 7596, 759690, 7597, 7598, 759900, 759991, 771100, 771140, 771210, 771220, 778000

© 2024 Turunen R et al. *JAMA Network Open*.

eTable 2. CHD subgroups classified according to Atlanta ICD-9 codes

Congenital heart defects subgroup	Atlanta ICD-9 codes
Complex	746, 7453, 7457, 745180,745190, 745300, 745420, 745610, 745700, 746800, 746810, 746820, 746887, 746888, 746889
TGA, transposition of great arteries	7451, 745100, 745110, 745120
LVOTO, left ventricular outflow tract obstruction	7463, 7464, 7365, 7466, 7467, 7471, 746300, 746400, 746401, 746480, 746481, 746490, 746500, 746505, 746580, 746581, 756600, 746601, 746700, 756900, 746901, 747100, 747110, 747190, 747200, 747210, 747220
RVOTO, right ventricular outflow tract obstruction	7452, 7460, 7461, 7462, 7473, 745200, 745210, 746000, 746010, 746020, 746021, 746080, 746081, 746090, 746100, 746105, 746106, 746181, 746200, 746830, 747130, 747200, 747300, 747310, 747320, 747380, 747390
Pulmonary venous anomalies	747420, 747430
Septal - others	745, 7450, 7456, 7456, 7458, 7459, 745000, 745010, 745400, 745620, 745630, 745680, 745690, 745800, 745900
VSD - ventricular septal defect	7454, 74548, 745480, 745481, 745482, 745483, 745484, 745485, 745486, 745487, 745488, 745490, 745491, 745492, 745493, 745494, 745495, 745496, 745498
ASD - atrial septal defect	745600, 7455, 745590, 745510, 745580
Thoracic arteries and veins	7472, 746885, 747200, 747215, 747240, 747250, 747270, 747280, 747290, 747330, 747340, 747380, 748685

© 2024 Turunen R et al. *JAMA Network Open*.

eTable 3. The ICD codes used in the definition of mother's diabetes[a]

**Definition**	**ICD codes**
T1DM	ICD-9: 250*B<br>ICD-10: E10, O24.0
T2DM / other type of diabetes	ICD-9: 250*A, 250*C, 250*X<br>ICD-10: E11, E12, E13, E14, O24.1, O24.2, O24.3
GDM	ICD-9: 6480A, 6488A<br>ICD-10: O24.4, O 24.9

Abbreviations: T1DM, type 1 diabetes; T2DM, type 2 diabetes or other diabetes; GDM, gestational diabetes.
[a]The ICD codes were used from MBR, the Care Register of Hospital Care and the Register of Special Reimbursement (special reimbursement for insulin).

© 2024 Turunen R et al. *JAMA Network Open*.

eTable 4. The prevalence of isolated CHDs[a] in the study population (n=620 751)

	No. (% of whole study population)
Isolated CHD	10 254 (1.65)
Complex	47 (0.01)
TGA	133 (0.02)
LVOTO	1105 (0.18)
RVOTO	1101 (0.18)
Pulmonary venous anomalies	36 (0.01)
Septal - others	411 (0.07)
VSD	6468 (1.04)
ASD	803 (0.13)
Thoracic veins and arteries	128 (0.02)

Abbreviations: CHD, congenital heart defect; TGA, transposition of great arteries; LVOTO, left ventricle outflow tract obstruction; RVOTO, right ventricle outflow tract obstruction; VSD, ventricular septal defect; ASD, atrial septal defect.
[a]See specified list of diagnose codes in eTable 1.

© 2024 Turunen R et al. *JAMA Network Open*.

eTable 5. The presence of maternal GDM and no DM according to BMI in mothers of children with and without isolated CHD[a]

**Mothers of children with isolated CHD**	**No DM (n = 8469)**	**GDM (n = 1524)**
BMI <18.5 kg/m^2, No. (%)	375 (95.4)	10 (2.5)
BMI 18.5-24.9 kg/m^2, No. (%)	5555 (89.7)	505 (8.2)
BMI 25.0-29.9 kg/m^2, No. (%)	1655 (76.0)	459 (21.1)
BMI ≥30 kg/m^2, No. (%)	711 (54.8)	534 (41.1)
**Mothers of children without isolated CHD**	**No DM (n = 520 460)**	**GDM (n = 85 503)**
BMI <18.5 kg/m^2, No. (%)	20815 (95.0)	1009 (4.6)
BMI 18.5-24.9 kg/m^2, No. (%)	342 982 (92.1)	27 527 (7.4)
BMI 25.0-29.9 kg/m^2, No. (%)	101 607 (78.2)	27 256 (21.0)
BMI ≥30 kg/m^2, No. (%)	44 247 (60.0)	28 326 (38.4)

Abbreviations: CHD, congenital heart defect, GDM, gestational diabetes; DM diabetes; BMI, body mass index.
[a]Data of mothers BMI status were missing in 12 454 (2.0%) cases.

© 2024 Turunen R et al. *JAMA Network Open*.

eTable 6. The comparison between individuals with missing and non-missing data and isolated CHD[a]

Characteristics	Isolated CHDs (n=10 254)	Healthy controls (n=610 497)	p-value
Missing data of mother's smoking, No. (%)	258 (2.52)	14 587 (2.39)	.40
Non-missing data of mother's smoking, No. (%)	9996 (97.5)	595 910 (97.6)	
Missing data of mother's BMI status, No. (%)	194 (1.9)	12 260 (2.01)	.43
Non-missing data of mother's BMI status, No. (%)	10 060 (98.1)	598 237 (98.0)	
Missing data of highest parental education level, No (%)[b]	471 (4.6)	30 177 (4.78)	.11
Non-missing data of highest parental education level, No (%)[b]	9783 (95.4)	601 777 (95.2)	

Abbreviations: No, number.
[a]The statistical significance was reached with p-value <.05. [b]Indicating the highest education level of either parent. Level of education categorized as level 1, level 2, and level 3 indicating low education, intermediate education, and high education.

© 2024 Turunen R et al. *JAMA Network Open*.

eFigure 1. Study flow chart

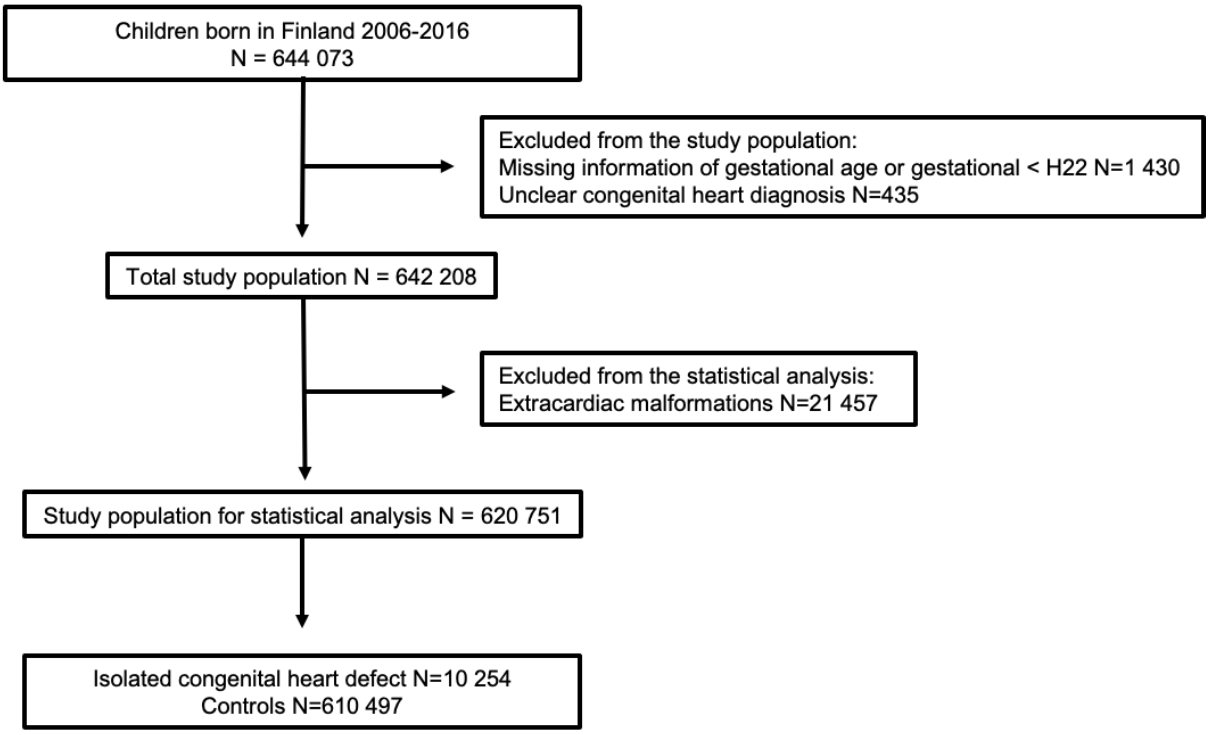

© 2024 Turunen R et al. *JAMA Network Open*.

eFigure 2. The prevalence of maternal diabetes and congenital heart defects during the study period

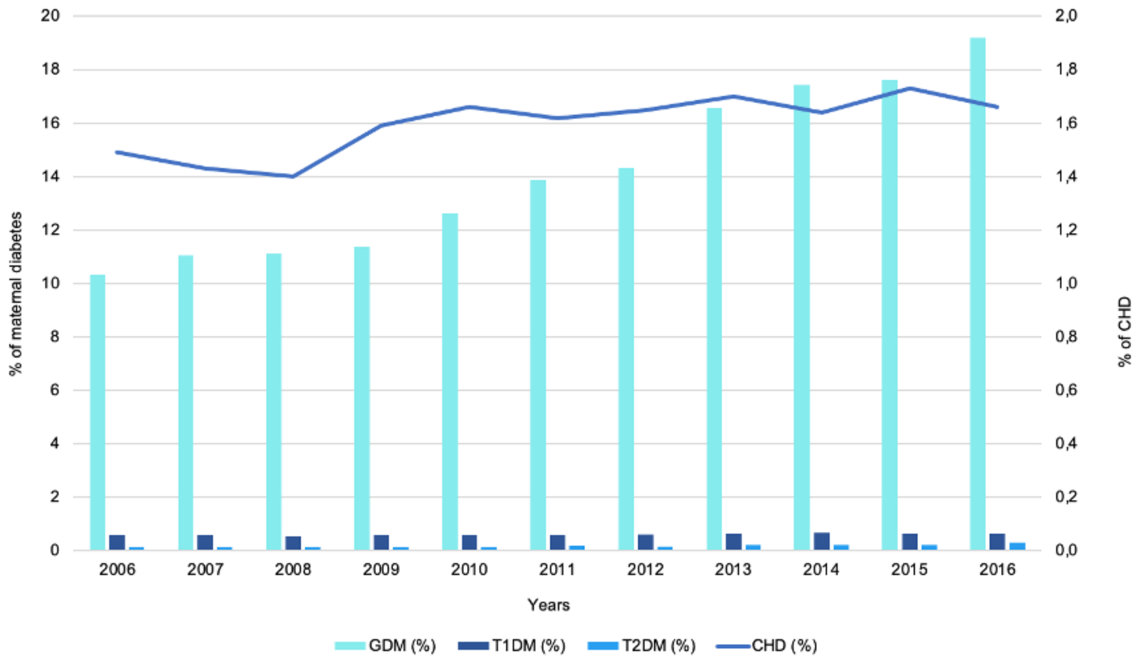

Abbreviations: GDM, gestational diabetes mellitus; T1DM, type 1 diabetes; T2DM, type 2 diabetes or other diabetes; CHD, congenital heart defect.

© 2024 Turunen R et al. *JAMA Network Open*.

eFigure 3. The prevalence of maternal obesity and congenital heart defects during the study period

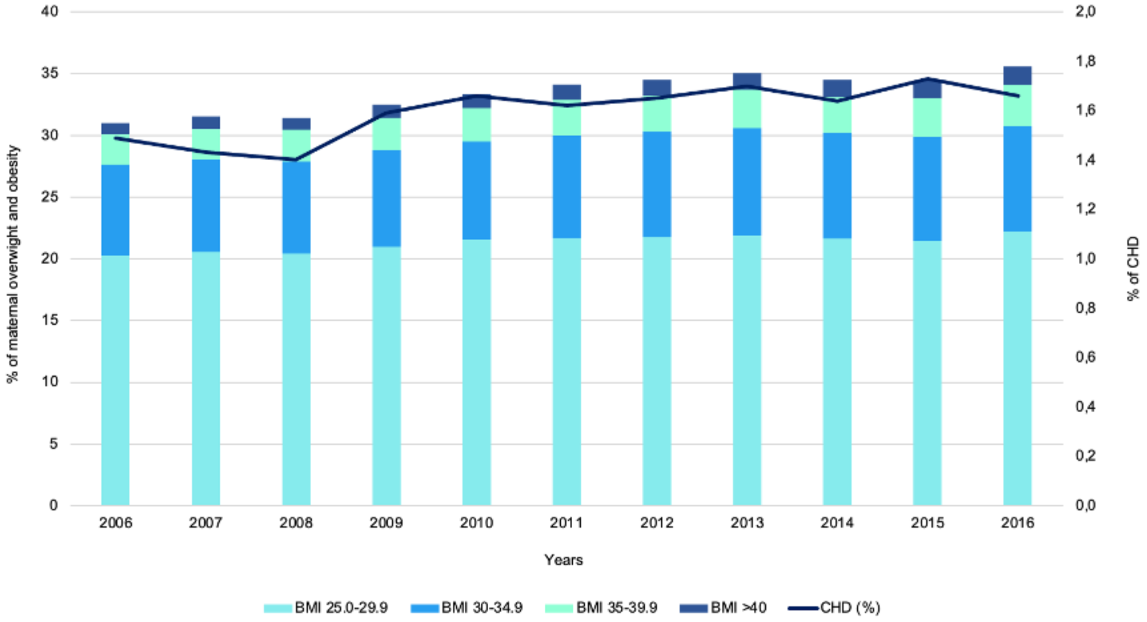

Abbreviations: BMI, body mass index; CHD, congenital heart defect.

© 2024 Turunen R et al. *JAMA Network Open*.

eFigure 4. The association between all CHD subgroups and maternal BMI and diabetes[a]

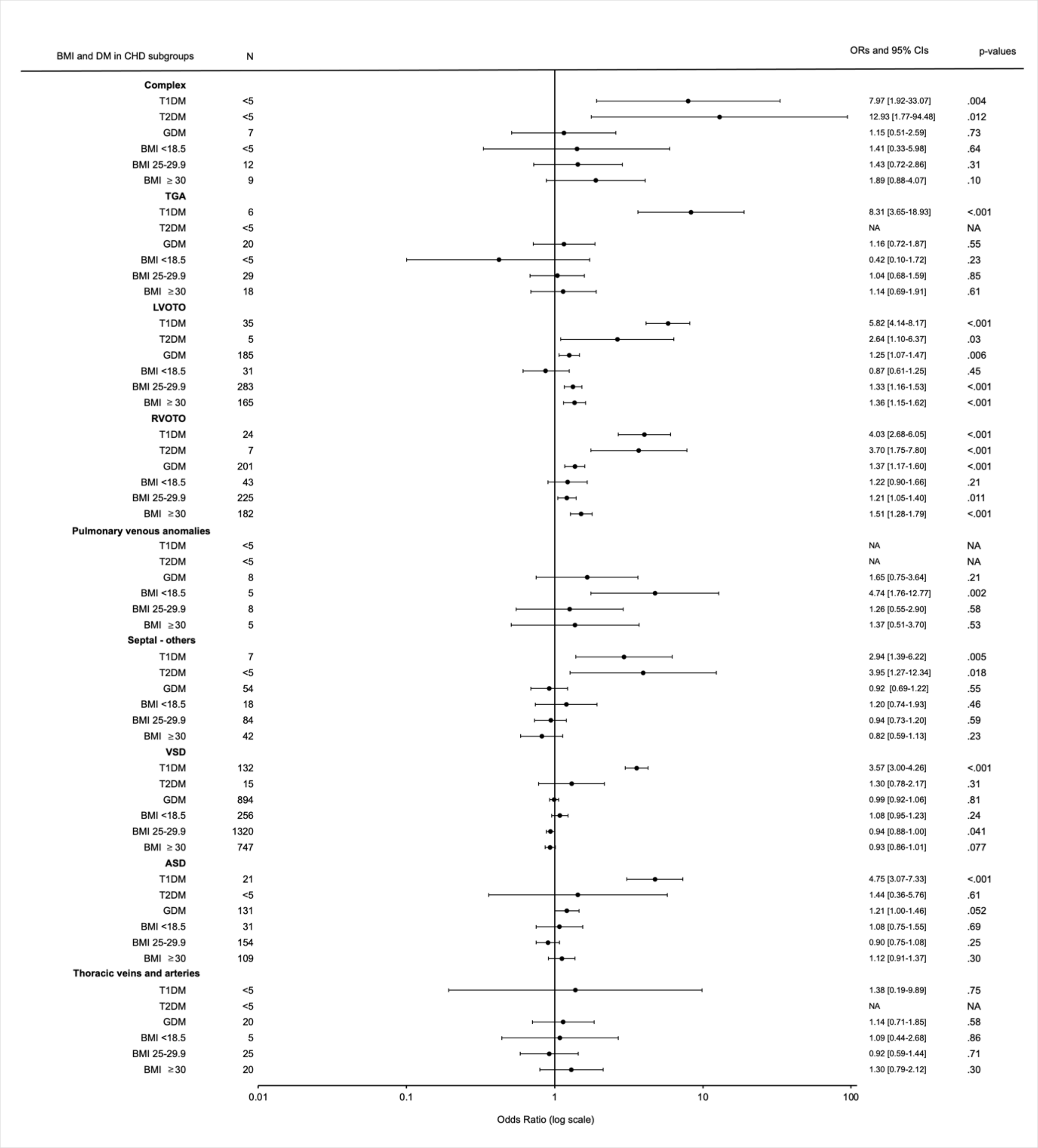

Abbreviations: CHD, congenital heart defects; BMI, body mass index; GDM, gestational diabetes mellitus; T1DM, type 1 diabetes; T2DM, type 2 diabetes or other diabetes; Na, not able to analyze; OR, odds ratio; CI, confidence interval; TGA, transposition of great arteries; LVOTO, left ventricle outflow tract obstruction; RVOTO, right ventricle outflow tract obstruction; VSD, ventricular septal defect; ASD, atrium septal defect.

[a]The analysis was adjusted to birth year of the child. Normal BMI (18.5-24.9) and no DM were used as a reference group. The statistical significance was reached with p-value <.05.

© 2024 Turunen R et al. *JAMA Network Open*.

eFigure 5. The association between maternal BMI and diabetes and all CHD subgroups using multivariable logistic regression analysis[a]

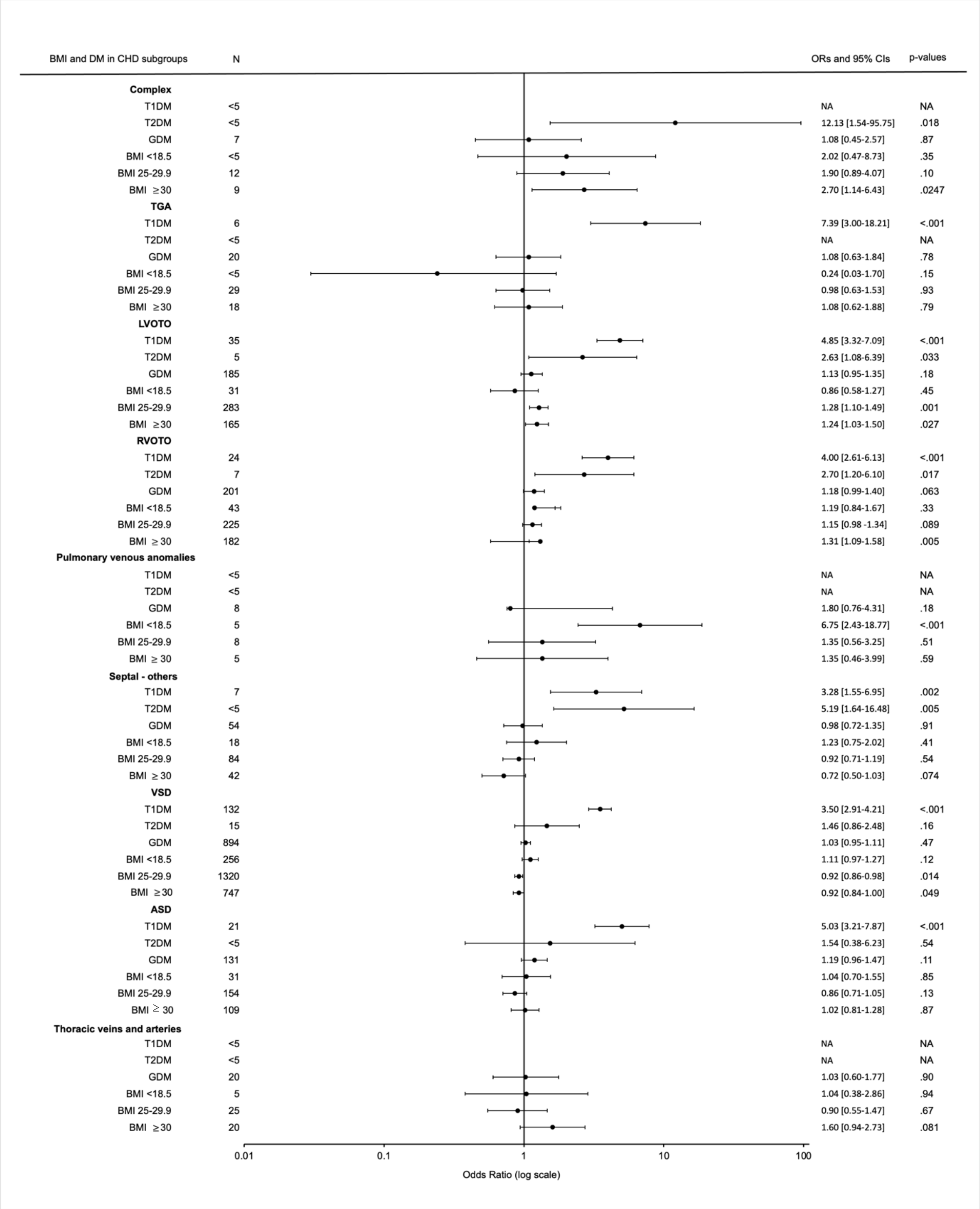

Abbreviations: CHD, congenital heart defect; BMI, body mass index; GDM, gestational diabetes mellitus; DMI, type I diabetes; DMII, type II diabetes; Na, not able to analyze; OR, odds ratio; CI, confidence interval; TGA, transposition of great arteries; LVOTO, left ventricle outflow tract obstruction; RVOTO, right ventricle outflow tract obstruction; VSD, ventricular septal defect; ASD, atrium septal defect. [a]The analysis was adjusted to maternal smoking, maternal age, child's birth year, first parity, highest parental education level. Normal BMI (18.5-24.9) and no DM were used as a reference group. The statistical significance was reached with p-value <.05.

© 2024 Turunen R et al. *JAMA Network Open*.
