# Supplementary material for: Maternal Diabetes and Overweight and Congenital Heart Defects in Offspring
Source: JAMA Netw Open. 2024 Jan 5;7(1):e2350579. doi: 10.1001/jamanetworkopen.2023.50579 (PMC10770771; doi:10.1001/jamanetworkopen.2023.50579)
Supplement: Supplement 2. — Data Sharing Statement [file jamanetwopen-e2350579-s002.pdf]

Data Sharing Statement

Turunen R. Maternal Diabetes and Overweight as Risk Factors Associated With Congenital Heart Defects in Offspring. *JAMA Netw Open*. Published online January 5, 2024. doi:10.1001/jamanetworkopen.2023.50579

Data
Data available: No

Additional Information
Explanation for why data not available: According to Finnish legislation researchers working outside the statutory register authority Finnish Institute for Health and Welfare can access register data for research purposes by applying for permission from the Finnish Social and Health Data Permit Authority Findata (findata.fi/en).
